# Acute Hepatitis C: Current Status and Future Perspectives

**DOI:** 10.3390/v16111739

**Published:** 2024-11-06

**Authors:** Massimo Fasano, Francesco Ieva, Marianna Ciarallo, Bruno Caccianotti, Teresa Antonia Santantonio

**Affiliations:** 1 Infectious Diseases Unit, PO Della Murgia “F. Perinei”, 70022 Altamura, Italy; 2 Department of Medical and Surgical Sciences, Section of Infectious Diseases, University of Foggia, 71122 Foggia, Italy; francesco-rosario@hotmail.com (F.I.); mariannaciarallo@virgilio.it (M.C.); brunocaccianotti@virgilio.it (B.C.); teresa.santantonio@unifg.it (T.A.S.)

**Keywords:** hepatitis C virus, modes of transmission, natural history, early phase of infection, DAA therapy

## Abstract

The hepatitis C virus (HCV) infection continues to represent a significant public health threat and is a leading cause of liver cirrhosis, liver cancer, and liver-related mortality. The World Health Organization (WHO) has set a goal for 2030: to eliminate HCV infection as a public health threat by reducing new HCV infections by 90% and mortality by 65%. The early phase of HCV infection represents a pivotal point in the evolution of hepatitis C. Despite a favourable course in the majority of patients, approximately 50–70% of individuals with recently acquired hepatitis C will develop a chronic infection, defined as the persistence of viremia for a period exceeding six months. The diagnosis and treatment of a recent HCV infection should facilitate engagement in multidisciplinary care, prevent the development and complications of chronic liver disease, and reduce ongoing transmission in key populations. Therefore, early treatment in the early phase of infection compared with deferring treatment until the chronic infection remains a valid approach in the era of direct antiviral agents (DAAs). This approach is supported by a cost-effectiveness analysis. The aim of this review is to synthesise the existing knowledge on the early phase of hepatitis C virus infection, with a particular focus on the current risk factors, natural history, therapeutic management, and future perspectives.

## 1. Introduction

The hepatitis C virus (HCV) is a single-stranded RNA virus belonging to the family Flaviviridae and the genus Hepacivirus, which is responsible for both acute and chronic hepatitis [1]. Since its discovery in 1989, the introduction of safe healthcare practices, the availability of simple diagnostic tests, and safe and effective antiviral therapies have made hepatitis C a preventable and treatable disease [2]. Despite these advances, HCV infection continues to represent a significant public health concern and is a primary cause of liver cirrhosis, liver cancer, and liver-related mortality. It is estimated that 58 million individuals worldwide are currently living with chronic HCV infection, with approximately 1.5 million new infections occurring annually [3]. In light of these findings, the World Health Organization (WHO) has set a goal for 2030: to eliminate HCV infection as a public health threat by reducing new HCV infections by 90% and mortality by 65%. In order to achieve these ambitious WHO targets, it is essential that at least 90% of infected individuals are diagnosed and that at least 80% of viremic individuals are treated [4].

The early phase of HCV infection represents a key point in the evolution of hepatitis C. The term “acute” hepatitis C traditionally denotes the initial phase of the HCV infection, defined as the first six months following initial exposure to the virus. This phase was historically significant due to the high probability of spontaneous viral clearance during this period. However, the term “acute hepatitis C” lacks a rigorous, universally accepted biological or clinical definition, and its use has become somewhat outdated with advancements in HCV diagnosis and treatment.

The aim of this review is to synthesise the existing knowledge on the early phase of hepatitis C virus infection, with a particular focus on the current risk factors, natural history, therapeutic management, and future perspectives.

### 1.1. Current Epidemiology, Routes of Transmission, and Risk Factors

The prevalence of HCV infection exhibits significant geographical variation, with notable discrepancies observed between and within countries globally. In Europe, it is estimated that there are approximately 13 million individuals who are chronic carriers of HCV, with a north–south gradient [3]. Published studies report varying incidence rates, which are likely to represent an underestimate of the true figure. This is because incident HCV infection is generally asymptomatic and therefore under-reported. The highest incidence rates are observed in the East Mediterranean and European regions, with 470,000 and 300,000 cases annually, respectively [4]. In the USA, the number of estimated new hepatitis C virus (HCV) infections decreased in 2022 for the first time after more than a decade of consecutive annual increases associated with the opioid epidemic. However, the number of estimated new HCV infections remained relatively high at 67,400 and did not meet the 2022 WHO’s target [5].

The most common route of transmission for HCV is parenteral exposure, with injection drug use and unsafe healthcare practices representing the most significant risk factors [6,7]. Other less common routes of transmission include vertical transmission from a HCV-infected mother to her baby and sexual transmission. The risk of sexual transmission of HCV is higher in men who have sex with men (MSM) and in people living with HIV (PLWH) or another sexually transmitted disease (Table 1).

The majority of new HCV infections continue to occur among people who inject drugs (PWID) [8]. Furthermore, an increasing incidence and prevalence of HCV infection have been reported over the past decade among HIV-positive or HIV-uninfected MSM, which is associated with an increase in sexual risk behaviour and recreational drug use [9,10,11].

A recent report from the Italian Surveillance System for Acute Viral Hepatitis (SEIEVA) indicates that the incidence of hepatitis C in 2022 has reached the level recorded in 2017. This follows a downward trend that has persisted for approximately a decade, with 55 new cases of acute hepatitis C and an incidence of 0.11 cases per 100,000 inhabitants. As in previous years, the most recent SEIEVA data identify healthcare-associated HCV exposure as the primary risk factor in Italy [12].

### 1.2. Diagnosis and Screening

Following exposure to HCV, the viral genome (HCV RNA) can be detected in the blood within one to two weeks, while antibodies directed against HCV proteins (anti-HCV) are not observed until 4 to 12 weeks after exposure. The standard testing algorithm for HCV includes initial testing for HCV-specific antibodies. Third-generation enzyme-linked immunosorbent assays (ELISAs) are currently employed for the detection of antibodies IgG to both structural and non-structural proteins of the HCV genome. The appearance of serum anti-HCV antibodies may be delayed and even absent in some patients, such as PLWH, haemodialysis patients, or patients undergoing organ transplantation who may fail to seroconvert to anti-HCV antibodies [13,14,15]. Therefore, in patients suspected of having incident HCV infection with a negative HCV antibody test, HCV RNA testing is required.

In contrast to other acute viral infections, there are no tests for the specific detection of IgM-type anti-HCV antibodies, which are used as a surrogate marker of primary infection.

**Figure 1:**
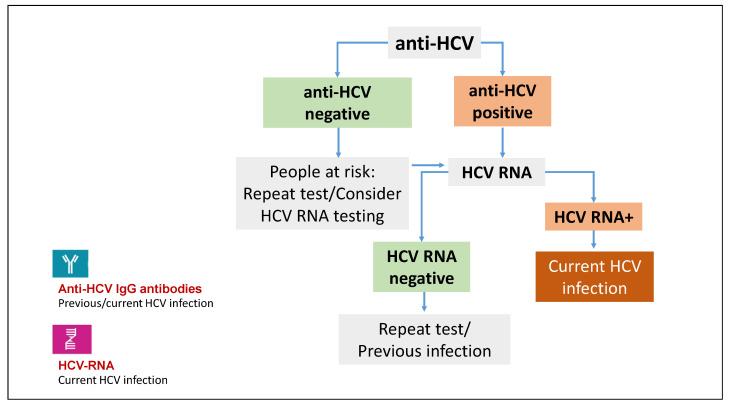
Acute HCV infection diagnostic algorithm.

In patients with detectable serum HCV antibodies, a highly sensitive molecular method such as real-time reverse transcription–polymerase chain reaction is recommended for the confirmation of current HCV infection (Figure 1).

In the interpretation of serological tests for the diagnosis of the early phase of HCV infection, it is crucial to consider that a positive test for both HCV antibody and HCV RNA indicates active HCV replication. However, this does not allow for the distinction between acute and chronic infections.

The most reliable laboratory evidence to support the diagnosis of a recent HCV infection is a positive HCV RNA test in the setting of a negative HCV antibody test (seronegative window period) or a documented anti-HCV seroconversion (positive HCV antibody test in a person who tested negative in the previous six months [6,16]). An alternative approach to diagnosing incident HCV infection is to consider at least two of the following criteria: (a) the presence of symptoms and signs compatible with acute viral hepatitis, including jaundice; (b) an elevated alanine aminotransferase (ALT) level greater than five times the upper limit of normal; (c) the presence of risk factors or a history of exposure to HCV; (d) the absence of other known causes of acute hepatitis, including hepatitis A virus, hepatitis B virus, hepatitis D virus in chronic hepatitis B infection, and autoimmune hepatitis. [6,16] (Table 2). In the context of hepatitis C, the necessity of HCV genotyping has been superseded by the advent of efficacious pangenotypic therapeutic regimens. Nevertheless, HCV genotyping can furnish invaluable epidemiological insights and can be useful in diagnosing HCV reinfection in high-risk populations.

A number of point-of-care (POC) tests are currently available for the diagnosis of hepatitis C, including antibody and RNA tests. The use of non-laboratory POC tests can facilitate the diagnosis of a recent HCV infection, the linkage to care, and the treatment of hard-to-reach high-risk populations such as drug users and prisoners.

In order to complete the diagnostic workup, patients with a recently acquired HCV infection must be screened for other viruses, such as HBV and HIV, which share the same mode of transmission as HCV. Furthermore, in patients with a sexually acquired recent HCV infection, an evaluation for concurrent sexually transmitted diseases is recommended [6].

### 1.3. Natural History

In general, an incident HCV infection is clinically silent, thereby evading clinical observation. Approximately 20% of patients exhibit symptoms, which may include fatigue, anorexia, nausea, vomiting, abdominal pain, and jaundice. The clinical course is typically benign, and hospitalisation is rarely indicated unless nausea and vomiting are severe. Acute liver failure is a rare complication of acute HCV infection, occurring in less than one percent of cases [16]. Despite a favourable course in the majority of patients, approximately 50–70% of individuals with acute HCV infection will develop a chronic infection, defined as the persistence of viremia for a period exceeding six months [16].

In a large cohort of patients with a well-documented diagnosis of recent HCV infection, we observed a resolution of the infection within 12 weeks from disease onset, in accordance with the findings of Gerlach et al. Subjects who remained viremic beyond this period were unlikely to experience spontaneous resolution and were, therefore, considered to have a chronic infection. [17,18].

It is noteworthy that transient suppression of viremia can occur in individuals with acute HCV infection, even in those who progress to a chronic infection. It can be reasonably concluded that a single undetectable HCV RNA test result is not sufficient to declare spontaneous clearance.

A number of host and viral factors, including the HCV genotype, HCV quasispecies diversity, HIV co-infection, sex, HLA, and age, appear to influence the outcome. In particular, young age, female sex, and the presence of symptoms have been associated with a spontaneous resolution of the infection [16,17].

Broad and multi-specific CD4+ and CD8+ T-cell responses have been associated with spontaneous clearance, whereas failure to mount a sustained T-cell response of sufficient magnitude has been associated with viral persistence [19,20,21,22]. However, the specific immunological features that predict clearance during the acute phase of infection remain poorly understood.

In patients with a spontaneous resolution of an acute HCV infection, a progressive reduction in anti-HCV antibody titers has been observed up to the complete clearance of HCV antibodies in some patients [17,23].

The current guidelines recommend that, following an initial diagnosis of the early phase of HCV infection, antiviral treatment should be initiated without delay, irrespective of whether the infection is expected to resolve spontaneously. The “test and treat” strategy can prevent loss of follow-up and reduce the risk of HCV transmission. In patients with recently acquired HCV infection, counselling is essential to prevent other causes of liver injury, including hepatotoxic drugs and alcohol use, and to reduce the risk of HCV transmission to others. Referral to an addiction medicine specialist is indicated for patients with an acute HCV infection related to substance use [6].

### 1.4. Treatment

The treatment of patients with incident HCV infection is recommended by national and international guidelines as a means of preventing complications associated with a chronic HCV infection and reducing transmission in high-risk populations [6,24,25].

The therapeutic regimens employed for the treatment of an acute HCV infection are identical to those recommended for chronic hepatitis C.

In the past, IFN-based therapies were used. Compared to chronic hepatitis C, treatment of the early phase of HCV infection has been characterised by higher cure rates, regardless of the genotype, and is achieved with a shorter duration of therapy (12 weeks) and even without the addition of ribavirin [26,27,28].

At the present time, IFN-based strategies have been superseded, and direct-acting antiviral (DAA) therapies represent the standard of care for both chronic and acute hepatitis C. DAAs can be classified into three principal categories based on their targets in HCV proteins. The first category comprises non-structural protein 3/4A (NS3/4A) protease inhibitors (PIs), which can impede HCV polyprotein processing. The second category encompasses NS5A inhibitors, which can inhibit viral replication and assembly. The third category consists of NS5B polymerase inhibitors, which can obstruct HCV RNA replication. The excellent outcomes achieved with DAA treatment in a chronic HCV infection have diminished the potential “efficacy advantage” of treatment in the early phase of HCV infection. However, diagnosis and treatment of a recent HCV infection should facilitate engagement in multidisciplinary care, prevent the development and complications of chronic liver disease, and reduce ongoing transmission in key populations. Therefore, treatment in the early phase of infection compared with deferring treatment until a chronic infection remains a valid approach in the DAA era and is supported by cost-effectiveness analysis [29].

High sustained virological response (SVR) rates have been reported in a number of small studies examining the efficacy and safety of DAA therapy [30]. However, the timing of treatment and optimal duration of DAA therapy in this setting are still undefined.

Treatment with a one-tablet co-formulation of sofosbuvir (NS5B polymerase inhibitor/ and ledipasvir (NS5A inhibitor) (SOF/LDV) administered for 6 weeks achieved a SVR in all treated patients (20/20) [31]. However, the same regimen yielded SVR rates by ITT and PP analyses of 77% and 88% in PLWH [32].

The extension of SOF/LDV therapy to 8 weeks resulted in sustained virologic responses in all 27 participants to the SWIFT-C cohort-2 study with HIV infections and acute HCV co-infections, suggesting that in this setting, a longer duration is required [33].

In recent times, the two more potent pangenotypic DAA regimens, glecaprevir (NS3/4A protease inhibitor)/pibrentasvir (NS5A inhibitor) (GLE/PIB) and sofosbuvir (NS5B polymerase inhibitor)/velpatasvir (NS5A inhibitor) (SOF/VEL) have been evaluated in clinical trials using shorter durations than recommended for the treatment of a chronic HCV infection.

In an open-label, single-arm, multicentre, international pilot study, Martinello et al. treated 30 adults (77% PLWH and 47% PWID) with a recent HCV infection (duration of infection < 12 months) with GLE/PIB for 6 weeks. A sustained virological response 12 weeks after stopping treatment (SVR12) was achieved in 90% (27/30) and 96% (27/28) of the intention-to-treat (ITT) and per-protocol (PP) populations, respectively. There was one case of relapse and two cases of non-virological failure (death, *n* = 1; loss to follow-up, *n* = 1). No treatment-emergent serious adverse events were observed [34].

In a more recent study, Martinello and colleagues evaluated the efficacy of four weeks of GLE/PIB in individuals with a recent HCV infection. The efficacy of a four-week treatment period was found to be inferior to that observed with longer treatment durations, specifically a minimum of six weeks. Indeed, SVR12 was achieved in 78% and 82% of the ITT and PP populations, respectively. Four cases of virologic failure were observed [35]. This short regimen of G/P for 4 weeks in adults with early HCV has also been evaluated in the ACTG 5380 study. SVR12 was achieved in 38 out of 45 (84%) participants (CROI) [36].

The efficacy of GLE/PIB therapy administered for 8 weeks is being further evaluated in an ongoing trial (EudraCT Number: 2020-005777-27).

An international, randomised, open-label, phase 3, non-inferiority trial (REACT trial) evaluated the efficacy of SOF/VEL administered for either 6 (*n* = 93, short duration) or 12 (*n* = 95, standard duration) weeks in patients with a recent HCV infection. In the ITT population, the SVR12 was 82% in the 6-week arm and 91% in the 12-week arm, with a lower response rate in the short-duration arm [37].

The HepNet Acute HCV V trial, which recruited 20 patients on HIV PrEP or opioid agonist therapy (OAT), extended the treatment duration of SOF/VEL to 8 weeks for acute HCV infection. Eighteen of the twenty patients (90%) achieved SVR12; the remaining two were lost to follow-up [38].

In view of these results, the AASLD/IDSA guidelines have recommended identical regimens for both acute and chronic hepatitis C, while the EASL guidelines recommend treating acute hepatitis C with the combination of SOF/VEL or with the combination of GLE/PIB for eight weeks until more definitive data are available.

There are currently no data available on the efficacy or cost-effectiveness of antiviral therapy for pre-exposure or post-exposure prophylaxis. Consequently, there is no indication of DAAs being used as post-exposure prophylaxis in the absence of documented HCV transmission [6,25].

### 1.5. HCV Reinfection

A spontaneous or treatment-induced resolution of incident hepatitis C does not confer sterilising immunity. As individuals with resolved acute infection may still be anti-HCV antibody positive, HCV RNA testing is essential to diagnose reinfection. The risk of reinfection following treatment for both acute and chronic HCV infection is increased in people who report persistent high-risk behaviour (such as continued injecting drug use). A recent meta-analysis revealed that the risk of HCV reinfection was significantly elevated among PLWH (3.76 per 100 PYs). Within this population, the incidence of reinfection among PLWH MSM and those with recent drug use was 6.01 and 5.49 per 100 years, respectively [39]. This highlights the need for post-treatment monitoring, rapid diagnosis of reinfection, and access to retreatment in this context.

The available data on the efficacy of DAAs in treating hepatitis C virus reinfection in patients who have previously achieved sustained viral response 12 following a course of DAAs are limited. The REACH-C cohort evaluated the efficacy of treatment with GLE/PIB, SOF/VEL, SOF/LDV, SOF/DCV, EBR/GZR, or sofosbuvir/velpatasvir/voxilaprevir (SOF/VEL/VOX) in 88 Australians with a HCV reinfection who had previously achieved SVR12 with a course of DAAs. All patients were people living with HIV, people who inject drugs, who were incarcerated, or who were on opioid agonist therapy. The SVR12 rate in the 56 patients with available outcomes was 95%, which was comparable to the 95% response rate observed in DAA-naïve patients. Eight patients were treated with the same regimen as their previous treatment, and all achieved SVR12 [40].

### 1.6. Vaccine Development

The advent of DAAs has greatly improved the efficacy of antiviral treatment, with the majority of treated patients achieving a cure. Nevertheless, the majority of infected individuals remain undiagnosed, and only a negligible number of those diagnosed receive antiviral treatment. Furthermore, re-exposure leads to reinfection, making control of HCV infection in high-risk populations a challenge. These considerations highlight the necessity for a safe and effective vaccine to prevent chronic HCV infection as a crucial element in efforts to eradicate the disease [41].

Incident HCV infection is known to resolve spontaneously in 10–45% of patients. This observation suggests that the immune response is capable of controlling the infection and preventing chronicity. It is, therefore, feasible to develop an effective HCV vaccine, although a vaccine that confers sterilising immunity is questionable because reinfection is possible after spontaneous clearance. Many vaccine approaches have focused on preventing chronic infection and, thus, liver disease, but the results have been disappointing. The high genetic diversity of HCV and the lack of suitable animal models present significant challenges for the development of a prophylactic HCV vaccine [42]. To date, only two vaccine candidates have been advanced to the stage of human trials. The initial vaccine formulation comprises the full-length recombinant E1/E2 glycoprotein of HCV GT1a, formulated with an oil-in-water adjuvant (MF59C.1). This vaccine was not pursued further since no subjects generated antibodies [43]. The second vaccination strategy uses a chimpanzee adenovirus vector (ChAd3) as the priming agent and a modified vaccinia Ankara (MVA) vector as the boosting agent, both encoding the HCV GT1b non-structural proteins. This vaccine was tested in a randomised, double-blind, placebo-controlled phase 1–2 trial in PWID. Although it induced strong HCV-specific T-cell responses and reduced peak HCV RNA levels, it did not prevent chronic HCV infection [44]. Recently, a bivalent pangenotypic prophylactic vaccine, consisting of a chimpanzee adenovirus vector (ChAdOx1) encoding conserved sequences across HCV GT1-6 and a modified HCV glycoprotein E2 with deletions of hypervariable regions (HVR) 1 and 2, has induced both neutralising antibody and CD4+ and CD8+ T-cell responses in mice experiments, but it has not been studied in humans [45].

## 2. Conclusions and Future Perspectives

Despite a decline in prevalence and incidence over recent decades, HCV infection continues to represent a significant global health threat and is not on target for elimination. The disease burden attributable to HCV is high among PWID and increasing among PLWH and MSM. Early diagnosis and increased treatment uptake in these key populations are essential to prevent the development and complications of chronic infection and to stop viral transmission. Screening protocols for recent HCV infection in these high-risk populations should be implemented, possibly using rapid or point-of-care diagnostics.

Treatment of the early phase of HCV infection is both safe and highly effective, preventing the development and complications of chronic liver disease and reducing ongoing transmission in key populations. The results of ongoing trials will determine the optimal timing of treatment initiation and duration of DAA therapy in recent HCV infection.

Many vaccine approaches have focused on the prevention of chronic infection and liver disease, but the results have been disappointing. Further exploration is therefore required to determine the role and feasibility of a HCV vaccine.

## Figures and Tables

**Table 1:** Current risk factors for HCV transmission and recommendations for testing.

At-Risk Population
**High risk**
People who inject drugs
Intranasal illicit drug users
Men who have sex with men
Blood transfusion recipients or transplantation before 1992
Persons on long-term hemodialysis
**Moderate risk**
High-risk sexual activity
Vertical transmission from mother to child
Persons who were ever incarcerated
HIV or HBV infection, Chronic liver disease, and/or chronic hepatitis, including unexplained elevated alanine aminotransferase (ALT) levels
**Low risk**
Occupational exposure
Sexual activity with long-term partners
Household contact

**Table 2:** Proposed criteria for diagnosis of incident hepatitis C.

**Primary criteria**	Serum HCV RNA-positive in a previously HCV RNA-negative patient Seroconversion from anti-HCV-negative to anti-HCV-positive
**Secondary criteria**	Elevated transaminase level > 5 times the upper limit of normal Known or suspected exposure to HCV within the preceding 6 months All other causes of acute liver damage are excluded Sudden onset of liver disease

## Data Availability

Not applicable.
